# Understanding *Ixodes ricinus* occurrence in private yards: influence of yard and landscape features

**DOI:** 10.1186/s12942-024-00380-9

**Published:** 2024-10-10

**Authors:** Anna Mazaleyrat, Jonas Durand, Irene Carravieri, Christophe Caillot, Cyril Galley, Sandrine Capizzi, Franck Boué, Pascale Frey-Klett, Laure Bournez

**Affiliations:** 1 ANSES, Nancy Laboratory for Rabies and Wildlife, 54220 Malzéville, France; 2 Tous Chercheurs Laboratory, UMR 1136 ‘Interactions Arbres Micro-Organismes’, Université de Lorraine, INRAE, Centre INRAE Grand Est-Nancy, 54280 Champenoux, France; 3 Centre Permanent d’Initiatives Pour l’Environnement (CPIE), Nancy Champenoux, 54280 Champenoux, France

**Keywords:** *Ixodes ricinus*, Tick, Occurrence, Yard, Landscape, Citizen science, Tick-borne disease prevention

## Abstract

**Background:**

Lyme borreliosis is the most frequent zoonotic disease in the northern hemisphere and is transmitted by ticks of the genus *Ixodes*. Although many people are bitten by ticks in private yards, our understanding of the factors associated with their presence in these areas remains limited. To address this gap, we used a citizen science approach to identify the local and landscape features associated with tick presence in yards.

**Methods:**

This study was conducted near Nancy, a city in northeastern France, from 2020 to 2022. Citizen scientists collected ticks in their yard on a single event (n = 185) and measured 13 yard features. Additionally, we computed 11 features related to the landscape composition and spatial configuration surrounding these yards. Using generalized linear mixed models, we determined the yard and landscape features associated with the presence of ticks and nymphal *Ixodes ricinus* (hereafter nymphs), the life stage, and species that mostly bite humans.

**Results:**

Despite a low density, ticks were found in 32% of the yards, including yards in urbanized areas. At the transect level, the likelihood of finding a nymph was nearly three times higher in transects shaded by vegetation compared to those in open areas, with no relationship between nymph occurrence and transect location or grass height. At the yard level, the occurrence of ticks and nymphs was related to both yard and landscape characteristics. Nymph and tick occurrence were more than twice as high in yards with signs of deer and a wood/brush pile compared to those without these characteristics, and increased with the connectivity of vegetation areas and the percentage of forest areas in the landscape.

**Conclusions:**

Our study reveals that private yards across an urbanization gradient are locations of tick exposure with tick presence linked to both yard and landscape factors. These findings emphasize the importance of public awareness regarding tick exposure in yards and provide crucial insights for future public health prevention campaigns.

**Supplementary Information:**

The online version contains supplementary material available at 10.1186/s12942-024-00380-9.

## Background

Ticks are hematophagous ectoparasites and can transmit many bacteria, viruses, and protozoa causing human and animal diseases [1, 2]. In the northern hemisphere, Lyme borreliosis is the most frequently reported zoonotic disease and an important economic burden for countries [3, 4]. For example, an estimated ≈ 476,000 and > 200,000 cases are diagnosed annually in the United States and in western Europe, respectively [5, 6], leading to an estimated annual cost of 345–968 million dollars in the USA [7] and 19–57 million euros for the Netherlands and Germany, respectively [3]. Lyme infection mostly manifests as an erythema migrans; disseminated forms may occur, causing neurologic, musculoskeletal, and cardiac complications [8].

The primary vectors of Lyme borreliosis are ticks from the *Ixodes ricinus* species complex (hereafter *Ixodes*), namely *I. ricinus* in Europe, *I. pacificus* in western North America, *I. scapularis* in eastern and mid-western USA and southern Canada, and *I. persulcatus* in Asia [9]. These ticks have three active life stages, larva, nymph, and adult, each requiring a blood meal to molt into the next stage (larva and nymph) or produce eggs (adult female) [10]. Although they can feed on a broad range of vertebrate hosts, including birds, small mammals, carnivores, and ungulates [11–14], in forests subadults mainly feed on birds and small- and medium-sized mammals, while adults are mainly fed by medium- and large-sized mammals (e.g. ungulates) [12, 13, 15]. In particular, deer are the most important maintenance hosts for these ticks, and their abundance/occurrence is known to drive tick populations [16, 17]. Identifying the drivers of *Ixodes* tick distribution and abundance is necessary to predict and mitigate tick exposure, thereby potentially reducing the risk of tick-borne diseases [18].

The distribution and abundance of ticks are linked to habitat suitability for both ticks and their hosts, which depend on complex interactions between abiotic (e.g. climatic conditions) and biotic conditions (host community and vegetation) playing differently at various spatial scales [19–21]. At the local scale, tick populations are also influenced by both abiotic and biotic conditions. Microclimatic conditions, impact the survival, development, and activity of *Ixodes* ticks [20, 22–28]. For example, low humidity and high temperatures usually have a detrimental effect on the survival of *Ixodes* ticks [22, 24, 25]. Microclimatic conditions are directly influenced by biotic habitat and soil characteristics such as vegetation structure (e.g. cover of near-ground vegetation) and composition (e.g. diversity of plant species), as well as soil texture. Besides buffering climatic extremes detrimental to tick survival [29, 30], trees and shrubs also create a litter layer with a humid microhabitat protecting ticks from desiccation during their off-host period [31–33]. Tick abundance also depends on the presence and abundance of their hosts [20], which are influenced by the structure and composition of vegetation (e.g. small mammals and birds [34, 35]), and interspecific interactions (e.g. competition and predation [36]). Vegetation affects the availability of food resources, shelters, breeding, or resting sites for hosts, while interspecific interactions can alter host movement and availability. Overall, several studies demonstrated a strong association between *Ixodes* ticks and woodland habitats (reviewed in: [31, 37]), likely reflecting both the higher availability of some propagation hosts (e.g. deer) and the more suitable microclimatic conditions in forest habitats compared to non-forest habitats.

At a broader scale, landscape composition (e.g. proportion of habitat types) and configuration (i.e. spatial arrangement of these habitats) can influence host availability through changes in the host community composition, host movement, and habitat use [19]. Landscape fragmentation, common in urban areas, results in a mosaic of patches of various sizes and land-use types, potentially impeding tick host movement [19] and isolating suitable habitats for ticks and their hosts from one another and from the source of large propagation hosts (e.g. deer in forests). In this context, ticks form a metapopulation, with subpopulations connected and reliant on each other for persistence. The degree of connectivity between subpopulations, enhanced by the tick hosts’ movements, determines whether tick subpopulations persist. Consistent with this explanation, previous studies in both urban and rural environments have demonstrated that connectivity between suitable patches for ticks and their hosts, as well as connectivity to populations of deer, are important for tick persistence (urban: [38–40]; rural: [41–43]). Several studies have reported an increased abundance or occurrence of *Ixodes* ticks with an increasing proportion of forest/tree canopy or the number of forest patches in the landscape [40, 43–47] and a decreasing proportion of agricultural areas and built-up and paved areas [39]. Considering the complex interplay between local and landscape habitat characteristics on ticks and their hosts, studies should examine both spatial scales concurrently (e.g. [44]).

Until now, most studies on tick ecology in Europe have primarily focused on forests, where ticks are most abundant [37]. Our understanding of the local and landscape features associated with tick occurrence in private yards thus remains limited (but see: Richter et al. in Germany [47] and Gregory et al. in the USA [38]). Nevertheless, this knowledge is fundamental to the prevention of tick-borne diseases as yards are important places for tick encounters and tick bites. Studies conducted in France, Belgium, and the Netherlands indicated that approximately 30% of reported tick bites occurred in yards [48–51]. The scarcity of research on tick populations in private yards can be primarily attributed to the challenge associated with tick sampling, as these private areas are not easily accessible to scientists. Citizen science, when members of the public collaborate with scientists to answer research questions, can help to overcome this challenge whilst likely increasing citizens’ tick-borne disease knowledge (as seen in a researcher—community partnership to promote Lyme disease prevention [52]). Citizen science has emerged as a powerful means of advancing tick research (reviewed in [53]) and is a valuable approach to gain insight into the complex ecology of ticks in yards.

TIQUoJARDIN, which translates to TICKinYARD in English, is a citizen science project conducted in northeastern France. This project aims to characterize the risk associated with the presence of ticks in private yards, encompassing the occurrence of ticks, their pathogens, and human exposure. The project also seeks to identify the local and landscape features related to this risk. To reach these objectives, citizen scientists followed a standardized protocol to collect ticks in their yard and completed surveys about the attributes of their yard and their tick bites. In this paper, we aim to characterize the presence of ticks in private yards and determine whether habitat characteristics at various scales, ranging from the local scale to the landscape scale, were associated with the occurrence of ticks. This study mainly focuses on *Ixodes ricinus,* i.e*.* the most widely distributed tick species in Europe, the primary vector of Lyme borreliosis, and a vector of many pathogens of public and veterinary health importance (e.g. tick-borne encephalitis, babesiosis, piroplasmosis, and anaplasmosis) [1, 54].

## Methods

### Study site

**Fig. 1:**
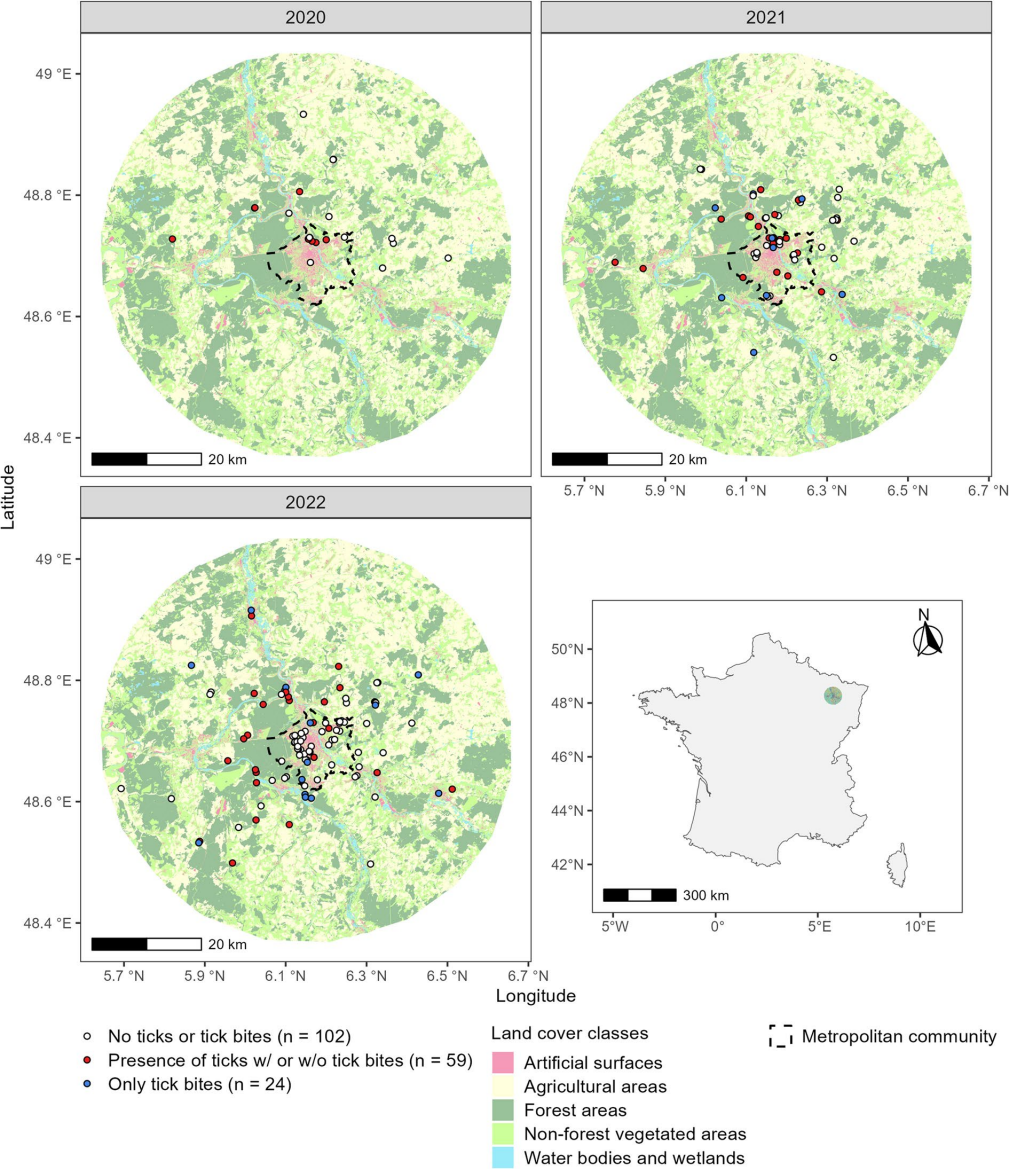
Location of the 185 sampled yards near Nancy, a city in the northeast of France. Tick sampling took place from May to mid-July 2020 (n = 26), 2021 (n = 59), and 2022 (n = 100) within a 35 km radius of Nancy. Land cover classes were modified from DataGrand Est [57]

This study took place within a 35 km radius of Nancy (48° 41′ 31.3′′ N, 6° 11′ 3.9′′ E), a city located in northeastern France (Fig. 1). Nancy (104,403 inhabitants) is part of a metropolitan community that covers 142 km^2^ and has ~ 255,000 inhabitants [55]. The climate is semi-continental with hot summers (mean daily maximum temperature during July is 25.8 °C) and cold winters (mean daily temperature during January is 2.6 °C) (1991–2020). The mean annual temperature is 11.0 °C and mean annual precipitation is 746 mm (1991–2020) [56]. The landscape is composed of agricultural areas (35% of the territory), non-forest vegetated areas (e.g. grassland and herbaceous areas, permeable surfaces in urban areas, and meadows), and forest areas (both representing 29% of the territory). Artificial surfaces (i.e. impervious surfaces only), and water bodies and wetlands cover 4% and 2% of the territory, respectively. In the metropolitan community, artificial surfaces cover 27% of the territory (based on data provided by DataGrand Est [57]).

### Tick sampling

A massive local media coverage of the project has been organized in 2020, 2021 and 2022 to recruit participating citizens to collect ticks in their yard. A yard was defined as a collective or an individual vegetated surface of at least 100 m^2^ adjacent or away from the dwelling maintained by the residents. Ticks were initially sampled in 213 private yards by the residents in 2020 (n = 28), 2021 (n = 73), or 2022 (n = 112) from May to mid-July, i.e. the period of peak activity of *I. ricinus* nymphs in the forest in northeastern France [58, 59]. Each yard was sampled once during the project. Due to missing yard features or key information to carry out our analysis, we only kept data from 185 yards (n = 26 in 2020, 59 in 2021, and 100 in 2022) (Fig. 1). Citizen scientists conducted the sampling following a standardized protocol using a “tick collection kit”, containing all the material to sample, identify, handle, and store ticks (see supplementary material S1 for details). Participants dragged a 1 m^2^ white cloth across the vegetation surface or over the leaf litter (e.g. [38]). To maximize tick detection probability, participants had to avoid tick sampling when vegetation was wet (ticks attach less well to a wet drag cloth) and sampling between 11 a.m. and 4 p.m. (*I. ricinus* activity decreases when temperature increases and/or relative humidity decreases [27, 28]). Participants had to sample 12 transects with each being 10 m long (~ 10 steps), four along property edges, and eight in the core of the yard. After each transect, they inspected the drag cloth (and their clothing to prevent tick bites and missing ticks) and transferred any tick (or what they thought might be a tick) into a labeled tube filled with 70% ethanol. Date and time of tick sampling were recorded. An expert later counted and morphologically identified the ticks to the species level and developmental stage according to Estrada-Peña et al. [60] and Pérez-Eid [61]. This study mainly focuses on *I. ricinus* nymphs due to the higher number of individuals collected compared to other stages and species (see result section) and that humans are predominantly bitten by *I. ricinus* nymphs [62, 63]. Given the low variability in the abundance of *I. ricinus* nymphs per yard (see result section), count data were converted into occurrence data.

To enhance tick presence assessment and to account for the single sampling event per yard, participants noted if they had been bitten (or likely bitten) by ticks in their yard during the three years preceding tick sampling.

### Habitat characteristics

To assess the influence of habitat characteristics on tick occurrence, we focused on three spatial scales: the transect scale, the yard scale, and the landscape scale.

#### Transect features

For each transect, citizen scientists recorded the transect location (edge vs core of the yard), grass height (< 10 cm, > 10 cm, and mixed, such as litter or various grass heights), shading conditions (partly under trees/bushes vs no tree branches/bushes above the transect), and approximate length (m), since it was not always 10 m long.

#### Yard features

We computed 13 yard features potentially influencing habitat suitability for *I. ricinus* or its hosts based mainly on the responses of participants to a questionnaire (Table 1). These included the presence/absence of a wood/brush pile, a stone wall or a pile of stone, a vegetable garden, a compost, an unmanaged long grass area, a bird feeder, and nut-producing trees (i.e. oaks, beech, hazel, and/or walnut). Participants also reported the presence of six fruit-producing group species and we computed the “number of fruit-producing species groups” per yard. Groups were based on the type of vegetation (i.e. trees vs. brushes and shrubs), and the period of harvesting (Table S1). Citizen scientists also indicated whether their yard was fully closed (preventing medium and large-sized mammals from entering the yard) or not. The mowing intensity was computed as the cumulative number of mowing events in spring, summer, and autumn based on participants’ reported lawn mowing frequency per season. Using the location of the yard, satellite images from Google Earth (including CNES/Airbus, GeoContent, and Maxar technologies images), and cadastral information [64], we computed the “vegetated surface of the yard” as the property surface excluding buildings (e.g. house) and unvegetated areas (e.g. pool and paved alleys). To characterize host presence, participants reported deer sightings or signs of their presence (e.g. feces and footprints) near their property (i.e. inside or within 5 m from the edges of their property). Participants also reported if they had a dog and if cats were present in their yard. Only the variable “presence of a dog” was considered due to the high prevalence of cats (96% of yards in 2021 and 2022, n = 159 yards) and missing information for 2020. The presence of free-ranging chicken was not considered due to the rarity of its occurrence (7% of the yards).

**Table 1:** Expected relationships between yard features computed and the occurrence of *I. ricinus* in yards

Yard features		Expected relationship with *I. ricinus* occurrence in yards	Explanation
Habitat suitability for tick hosts	Presence of A wood/brush pile A stone wall or a pile of stone A vegetable garden A compost A bird feeder	+	These attributes can provide a shelter (i.e. the presence of a wood/brush pile, a stone wall or pile of stone) or food for tick hosts (i.e. the presence of a vegetable garden, a compost, and a bird feeder) leading to an increase in host richness or abundance [97, 98, 107]. Previous studies have thus shown a positive relationship between the presence of these features and tick occurrence in yards or recreational sites [38, 94, 95]
	No. of fruit-producing species groups	+	Fruit or nut-producing trees can provide food for tick host species (mainly small mammals and birds) influencing their abundance (e.g. [108–110]). Host species abundance may in turn influence tick abundance [108]. The abundance of *I. ricinus* nymphs in pasture has been shown to be positively related to the presence of fleshy fruit trees at pasture edges [87]
	Presence of nut-producing trees		
	Vegetated surface of the yard (m^2^)	+	The relative abundance of tick host species can increase with yard size [97] and tick abundance has been shown to increase with the vegetated surface of the yard [111]
Yard accessibility and presence of tick hosts	Yard closure	–	Yard fencing can reduce the diversity and abundance of host species by preventing medium and large-sized mammals from entering a yard [98, 112, 113]. Full fencing around the yards has been shown to decrease the odds of finding *I. scapularis* [38] (but see: [95])
	Signs of deer	+	Deer play a key role in the persistence of *I. ricinus* populations as adult ticks mainly feed and copulate on deer [12]. Previous studies showed that the density of *I. ricinus* nymphs in forests was positively associated with deer presence [17]. In green spaces (mainly parks), *I. ricinus* density increased with the connectivity to a known population of roe deer (*Capreolus capreolus*) [39]
	Presence of a dog	+/−	Dogs can bring back ticks into the yard after a walk outside the yard (e.g. in park [114]) or serve as hosts for feeding ticks in yards. Alternatively, a free dog in a yard can prevent some hosts from entering the yard [112]
Habitat suitability for ticks	Mowing intensity	–	Increasing mowing intensity and the presence of an unmanaged long grass area are expected to decrease and increase the availability of moist microhabitats for ticks, respectively. As low humidity and high temperatures usually have a detrimental effect on the survival of *Ixodes* ticks [22, 24, 25], these features are expected to affect tick occurrence or abundance. However, the influence of mowing intensity and the presence of an unmanaged long grass area on tick abundance or occurrence has been seldom tested appropriately. A study showed that a single mowing event did not affect the abundance of *I. scapularis* and *Dermacentor variabilis* on recreational hiking trails [93], while another demonstrated that tick density was not associated with grass height in pasture edges [87]
	Presence of an unmanaged long grass area	+	

#### Landscape features

We used land cover data obtained from high-resolution orthophotos [57] to classify land cover into five classes: 1) artificial surfaces (i.e. impervious surfaces only), 2) non-forest vegetated areas (e.g. grassland and herbaceous areas, permeable surfaces in developed areas, and abandoned agricultural areas), 3) forest areas (e.g. deciduous forest, clearcuts, and young plantations), 4) agricultural areas (e.g. annual and perennial crops), and 5) open water and wetlands. Subsequently, we derived eleven features describing the landscape composition and spatial configuration that can influence host availability and the availability of suitable habitats for ticks (Table 2). The six spatial configuration variables were the density of forest patches, the shortest distance to a forest patch, the effective mesh size of vegetation areas (i.e. combining forest and non-forest vegetated areas), the effective mesh size of vegetation and agricultural areas combined, the total edge density, and the edge density of vegetation areas. The effective mesh size (MESH) is a fragmentation index and represents the average size (ha) of areas (i.e. vegetation areas or vegetation and agricultural areas combined) that an organism is connected to in a landscape starting from a randomly chosen point [65]. The more barriers in the landscape (e.g. artificial surfaces), the less vegetation areas organisms will have access to, and the lower the effective mesh size. Since we do not have specific knowledge about which species contribute to tick presence in yards and their movements, particularly in urban environments, we computed these variables (except for the shortest distance to a forest patch), within buffer sizes of 300, 400, and 500 m to maximize variability and avoid missing values. Indeed, when the landscape consists of only one vegetation patch, the MESH cannot be computed. To describe the landscape composition, we computed five variables, the percentage of artificial surfaces, non-forest vegetated areas, forest areas, and agricultural areas within the three buffer sizes, and the percentage of vegetation areas within a 50-m buffer, to characterize the immediate environment surrounding the yard (Table 2).

**Table 2:** Expected relationships between landscape features and the occurrence of *I. ricinus* in yards

Landscape features		Expected relationship with *I. ricinus* occurrence in yards	Explanation
Landscape composition	Percentage of		
	artificial surfaces	–	Due to the lack of a litter layer and unsuitable microclimatic conditions, artificial surfaces are unsuitable habitats for ticks. Moreover, they can impede the movement of hosts, especially large-sized mammals which are key hosts of adults *I. ricinus* [12], and isolate the yard from putative sources of ticks and hosts such as forests. Consistently, densities of *I. ricinus* (all stages) in green spaces in Belgium decreased with increasing urbanization (i.e. the proportion of built-up and paved areas [39])
	agricultural areas	+/−	On one hand, crops are less suitable habitats for ticks (compared to forests) due to the lack of a litter layer and less suitable microclimatic conditions [115]. On the other hand, agricultural practices can alter host movements, and habitat use, with some mammals being more (or less) abundant in crops compared to mature forests [116, 117]. Previous studies showed that the densities of *I. ricinus* larvae and adults (but not nymphs) in green spaces in Belgium decreased with the percentage of agricultural areas [39]
	non-forest vegetated areas	+	Non-forest vegetated areas (e.g. parks and pasture) can be suitable habitats for ticks [39, 87] and are used by some tick hosts [116, 118]. In urban green spaces (e.g. parks, natural and amenity green spaces), the density of *I. ricinus* nymph was positively associated with the proportion of vegetation areas in the landscape (i.e. urban forests, open green spaces, and green corridors such as wildflower verges and hedgerows [81]), while in forests it decreased with the percentage of pastures in the landscape [44]
	vegetation areas (forest and non-forest vegetated areas combined)		
	forest areas	+	Given that the abundance of some host species increases with the percentage of forest areas in the landscape [45] and that *I. ricinus* is most abundant in forests than in other habitat types [37], many studies reported an increase in *I. ricinus* abundance or occurrence in forests or green spaces with the proportion of forests or the number of forest patches [43–47]. The density and occurrence of nymphal *I. ricinus* in green spaces were negatively related to the distance to woodland [81]
Landscape configuration	Density of forest patch (number.100 ha^−1^)		
	Min. distance to the forest (m)	−	
	Effective mesh size (MESH, ha) of vegetation areas of combined vegetation and agricultural areas	+	Fragmentation can alter host availability through changes in the host community composition, host movement, and habitat use. Highly fragmented habitats may hold populations of smaller and/or more mobile tick host species (e.g. rodents and birds), but not of larger animals (e.g. deer) [19]. Previous studies in both urban environments [39] and rural environments [41–43] have demonstrated that connectivity between suitable patches for ticks and their hosts, as well as connectivity to source populations of large propagation hosts (i.e. forest), are important for *I. ricinus* persistence
	Edge density (m. ha^−1^) of landscape of vegetation areas	+/−	On one hand, some mammal species can be more abundant at habitat edges, i.e. zones of transition between adjacent ecological systems, than in the habitat interior (e.g. forest edges) [119, 120]. On the other hand, increasing edge density implies increased habitat fragmentation, which could be detrimental to the movement of tick host species. The abundance of adult *I. ricinus* has been shown to increase with the forest edge density, while that of nymph was not related to the edge density of forest [43]

### Meteorological data

To account for variation in meteorological conditions during sampling, we computed the average of daily saturation deficit per yard during the five days preceding tick sampling. Indeed, the saturation deficit affects the questing activity of *I. ricinus* [27, 28, 66] and could thus affect the likelihood of finding ticks. For each yard and each of the five days preceding tick sampling, we computed the daily saturation deficit (mmHg) using the daily average of relative humidity (%), the daily average of temperature (°C), and the formula provided by Randolph and Storey [66]. We then averaged those five values. For each yard, we used hourly temperature and relative humidity data from the nearest weather station (mean distance between yards and the weather stations: 7770 m, range: 724 − 26,835 m, n = 185) [56].

## Statistical analysis

In this paper, we investigated the relationships between the occurrence of *I. ricinus* nymphs and habitat characteristics at transect, yard, and landscape levels. The low detectability of ticks by drag sampling when their abundance is low [67], coupled with the fact that ticks may not always be present in a yard, may mask or bias statistical relationships between tick occurrence and environmental variables. We thus examined how incorporating tick bites in the assessment of tick occurrence could influence the relationships between tick occurrence and environmental variables. To do so, we explored the relationships between the occurrence of ticks (0 when no ticks were collected during tick sampling, otherwise 1), and the corrected occurrence of ticks (0 when no ticks were found during tick sampling and none of the household members reported tick bites in the yard during the three years preceding tick sampling; otherwise, 1) and yard and landscape features. For all the following analyses, we used generalized linear mixed models (GLMMs) with a binomial error distribution (logit link).

### Analysis of the occurrence of nymphal I. *ricinus* at the transect-level

We assessed the relationships between transect characteristics and the presence of *I. ricinus* nymphs at the transect-level. In this analysis, we only considered yards where nymphs were found, to only include suitable conditions for tick presence and avoid background noise due to unsuitable landscape characteristics. Fixed effects included transect location (edge *vs* core), grass height (< 10 cm, > 10 cm, and other), and shading (shaded *vs* not shaded). The length of the transect, log-transformed, was included as an offset variable to adjust models for differences in sampling effort among transects. The offset variable makes model adjustments with its regression coefficient being fixed at 1 [68]. To account for variations in sampling conditions between years and repeated measures within yards, we included the yard ID nested in the sampling year as a random effect. For this analysis, we only kept data from yards for which information was available for all transects (i.e. length, location, grass height, and shading), i.e. 434 transects (n = 48 in 2020, 226 in 2021, and 160 in 2022) across 37 yards (4 in 2020, 19 in 2021, and 14 in 2022).

### Analysis of the occurrence of nymphal *I. ricinus* and ticks, and the corrected occurrence of ticks at the yard level

To assess the relationships between yard and landscape features, and the occurrence of *I. ricinus* nymphs, ticks, and the corrected occurrence of ticks, we used occurrence data at the yard level. The total length of transects sampled per yard (log-transformed) was included as an offset variable, to account for variations in sampling effort among yards, and the sampling year as a random effect. Fixed effects included 13 yard features (Table 1), 11 landscape features (Table 2) (see below for the selected buffer size), and the 5-day average of daily saturation deficit to account for temporal variability of meteorological conditions. These analyses rely on data from 185 yards (n = 26 in 2020, 59 in 2021, and 100 in 2022).

For each of the three response variables, we built models that included different combinations of yard and landscape features. To avoid collinearity issues, we did not build candidate models that included variables with an absolute value of the Pearson correlation coefficient greater than 0.6. Model selection was based on the Akaike information criterion (AIC) and the parsimony principle*.* We selected models within ΔAICc < 2, as there is substantial evidence to support them as the best models to explain the observed patterns in the data [69]. If multiple models were chosen, the ones with fewer variables was preferred. To gain insight into the respective influence of yard and landscape features, the same model selection procedure was applied using combinations of either yard or landscape features. Model accuracy was evaluated using the AIC, AUC, sensitivity, and specificity. For each best model, the ROC curve was used to compute the AUC. The Youden’s index [70] (sensitivity + specificity – 1) was computed for all points of the ROC curve. The highest index value was then used to select the optimal cut-off value that optimizes both sensitivity and specificity. A predicted occurrence greater than the cut-off threshold was assigned as tick (corrected or not) or nymphs were present. The sensitivity and specificity were computed using the optimal cut-off.

Before exploring the relationships between environmental factors and the three response variables, we identified the optimal buffer size at which the relationships between landscape features and the occurrence of ticks were the strongest (see also [40]). Indeed, variables computed with different buffer sizes were highly correlated (Fig. S1) which can cause multicollinearity. For each of the three buffer sizes, we built candidate models that included as fixed effects different combinations of the nine variables computed at various buffer sizes. We included the total length of transects sampled per yard (log-transformed) as an offset variable and the sampling year as a random effect. The best model for each buffer size was selected based on the AIC and the parsimony principle, as previously described. We then compared the AIC of the three best models and chose the buffer size at which the AIC was the lowest. A buffer size of 500 m was the best fit for tick occurrence (AIC = 205.71 compared to AIC = 206.71 and AIC = 207.50 for buffers of 300 and 400 m, respectively). As a result, we kept landscape features computed within a 500 m buffer.

All analyses were carried out in R v 4.3.0 [71] using the packages g*lmmTMB* [72] for GLMMs and *MuMIN* [73] for model selection (dredge function). Collinearity was assessed using the variance inflation factor (VIF) (package *performance*, [74]) and none was detected. The DHARMA package [75] was used to check for spatial autocorrelation (Moran’s I test) and for patterns in the residuals using simulation-based standardized residuals. No spatial autocorrelation or patterns in residuals were detected. The package *cutpointr* [76] was used to find the optimal cutpoint and compute the accuracy metrics (i.e. AUC, sensitivity, and specificity) using these thresholds. The 95% CI of the accuracy metrics was computed using the package *pROC* [77]. Predicted odd ratios and their confidence intervals were obtained with the *sjPlot* package [78].

## Results

### Description of sampled yards

Following the classification of the territory according to its degree of urbanization [79, 80], 33% of sampled private yards with complete data (n = 185) were in densely populated urban areas (cities), 29% in urban areas of intermediate density (towns and suburbs), and 38% in thinly populated rural areas. These yards were almost exclusively private individual yards adjacent to or surrounding dwellings (96.2%). Others were individual yards away from the dwelling (2.2%), or collective yards (1.6%). Yards differed in their yard and landscape features (Table 3). On average, yards had a vegetated surface of 953 m^2^ (SD = 1246) and were located 310 m from a forest patch (SD = 318). The three most common yard features were the presence of a compost, the presence of a vegetable garden, and the presence of a bird feeder (83%, 76%, and 71% of the yards, respectively). Additionally, over half of the yards had a wood/brush pile, a stone wall or pile of stone, or nut-producing trees while having a dog, a fully closed yard, and signs of deer were less common. On average participants mowed their lawn approximately twice a month in spring, summer, and autumn. Within a 500 m buffer surrounding the yard, the mean percentages of artificial surfaces and non-forest vegetated areas were 28% (SD = 17) and 50% (SD = 12), respectively, while the mean density of forest patches was 4.0 patches.100 ha^−1^ (SD = 3.7) (Table 3).

**Table 3:** Variation of the yard and landscape features in private yards (n = 185)

Continuous variables	Mean ± SD	Range	Freq. of occurrence of *I. ricinus* nymphs in yards when the feature is	
			Below average (%)	Above average (%)
Mowing intensity	18.3 ± 9.5	2–36	22	26
Vegetated surface of the yard (m^2^)	953 ± 1246	91–8846	19	35
No. of fruit-producing species groups	3.4 ± 1.8	0–6	13	34
5-day avg. daily saturation deficit (mmHg)	4.7 ± 2.0	1.3–11.7	26	22
Percentage of artificial surfaces^500^	28 ± 17	2–70	32	12
Percentage of agricultural area^500^	9 ± 13	0–51	28	16
Percentage of forest areas^500^	10 ± 13	0–57	14	43
Percentage of non-forest vegetated areas^500^	50 ± 12	21–80	27	20
Percentage of vegetation areas^50^	59 ± 16	11–100	14	33
Edge density of landscape^500^ (m. ha^−1^)	354 ± 118	82–652	29	18
Edge density of vegetation areas^500^ (m.ha^−1^)	346 ± 131	65–651	30	17
Density of forest patch^500^ (number. 100 ha^−1^)	4.0 ± 3.7	0.0–18.3	20	30
MESH of vegetation areas^500^ (ha)	9.6 ± 10.2	0.2–60.6	15	44
MESH of combined vegetation and agricultural areas^500^ (ha)	14.6 ± 13.7	0.2–74.5	16	35
Min. distance to the forest (m)	310 ± 318	0–1856	31	11

Dichotomous variables	Percentage of yards with the feature (number)	Freq. of occurrence of *I. ricinus* nymphs in yards	
		With the feature (%)	Without the feature (%)
Compost	83% (153)	27	9
Vegetable garden	76% (140)	25	20
Bird feeder	71% (132)	22	28
Nut-producing trees	61% (112)	31	12
Stone wall or pile of stone	57% (105)	22	26
Wood/brush pile	55% (102)	33	12
Unmanaged long grass area	41% (75)	29	20
Dog	22% (41)	20	25
Signs of deer	20% (37)	57	16
Yard closure (fully closed)	18% (34)	18	25

These variables were used to explore the relationships between environmental factors and the occurrence of nymphal *I. ricinus*, ticks, and the corrected occurrence of ticks. Variables were computed within a 500-m (^500^) or a 50-m buffer (^50^) from the edges of the property. MESH: effective mesh size. Vegetation areas are composed of forest areas and non-forest vegetated areas

### Tick collection and tick bites

Citizen scientists sampled a total of 2030 transects in 185 yards. As indicated in the protocol, tick sampling took place at the appropriate time of day (i.e. before 11 a.m. or after 4 p.m.) 93% of the time. Temperature and relative humidity during sampling events that did not take place within the appropriate time of the day (mean ± SD; temperature: 20.3 ± 4.1 °C; relative humidity: mean ± SD: 51.9 ± 12.3%) was similar to those carried conducted at the appropriate time of the day (temperature: 20.4 ± 4.7 °C; relative humidity: 55.7 ± 16.2%). On average, citizen scientists sampled 11 transects per yard (SD = 2.0, range 4–12), covering an average area of 109 m^2^ (SD = 22.7, range 36–200 m^2^). 84% of citizen scientists sampled at least 10 transects. Yards with fewer than ten transects had a mean vegetated surface of 339 m^2^ (SD = 295), compared to a mean of 1072 m^2^ (SD = 1323) for yards with at least ten transects. Citizen scientists, collected 501 ticks (112 larvae, 365 nymphs, and 24 adults) belonging to four species: *Ixodes ricinus*, *Ixodes frontalis*, *Dermacentor marginatus*, and *Dermacentor reticulatus*. *Ixodes ricinus* was by far the most collected species (96% of the ticks collected) and *I. ricinus* nymphs (hereafter “*nymphs*”) represented 71% of the collected ticks (Table 4). Ticks occurred in 8.6% of the transects and 32% of sampled yards (Table S2). At least one nymph was found in 24% of yards (Table S2), with an occurrence of 18% in yards in densely populated urban areas, 30% in urban areas of intermediate density, and 24% in thinly populated rural areas. Other species and life stages occurred in less than 6% of the yards, except for *I. frontalis* larvae in 2020 (15% of the yards, 4 yards) and *I. ricinus* male in 2021 (8% of yards, 5 yards) (Table S2). The average density of nymphs was 1.6 individuals per 100 m^2^ (SD = 6.3, range 0–62.9). In yards with nymphs (n = 44), the median density was 2.5 nymphs per 100 m^2^ (mean: 6.7, SD = 11.7, range 0.8–62.9).

**Table 4:** Frequency of each tick species, stage, and sex (number collected) per sampling year

Species	Stage and sex	2020	2021	2022
*Ixodes ricinus*	Larva	52% (96)	2% (4)	5% (4)
*Ixodes ricinus*	Nymph	41% (75)	91% (224)	81% (59)
*Ixodes ricinus*	Adult female	1% (2)	1% (3)	3% (2)
*Ixodes ricinus*	Adult male	1% (2)	3% (7)	3% (2)
*Ixodes frontalis*	Larva	4% (8)	0% (0)	0% (0)
*Ixodes frontalis*	Nymph	0% (0)	1% (3)	5% (4)
*Dermacentor marginatus*	Adult female	0% (0)	0% (0)	1% (1)
*Dermacentor reticulatus*	Adult female	0% (0)	1% (2)	0% (0)
*Dermacentor reticulatus*	Adult male	0% (0)	1% (2)	1% (1)
	Total	100% (183)	100% (245)	100% (73)

Tick collection took place in 185 private yards from May to mid-July 2020 (n = 26), 2021 (n = 59), and 2022 (n = 100) within a 35 km radius of Nancy, a city located in the northeastern of France

Among households (n = 185), 29% indicated that at least one family member had been (very likely) bitten by ticks in their yard in the last three years. In the last three years, 48% of family members reported being bitten by ticks and 18% reported being bitten by ticks in the yard (n = 485, data only available for 2021 and 2022). When corrected by tick bites in the yard, ticks potentially occur in 45% of the yards (35% in 2020, 56% in 2021, and 41% in 2022).

### Relationships between transect features and the occurrence of nymphal *I. ricinus* at the transect-level

In yards with nymphal *I. ricinus* and for which information was available for all transects (n = 434 transects in 37 yards), nymphs occurred in 27% of the transects (SD = 24). In these yards, nymphs occurred in 34% of shaded transects compared to 17% of unshaded transects (Table S3). The likelihood of finding at least one nymph increased by 2.75 (95% CI 1.52–4.97) when the transect was shaded by vegetation compared to not shaded transect. Grass height and the location of the transect (edge *vs* core) were not related to the likelihood of observing nymphs.

### Relationships between yard and landscape features, and the occurrence of *I. ricinus* nymphs, ticks, and the corrected occurrence of ticks

**Fig. 2:**
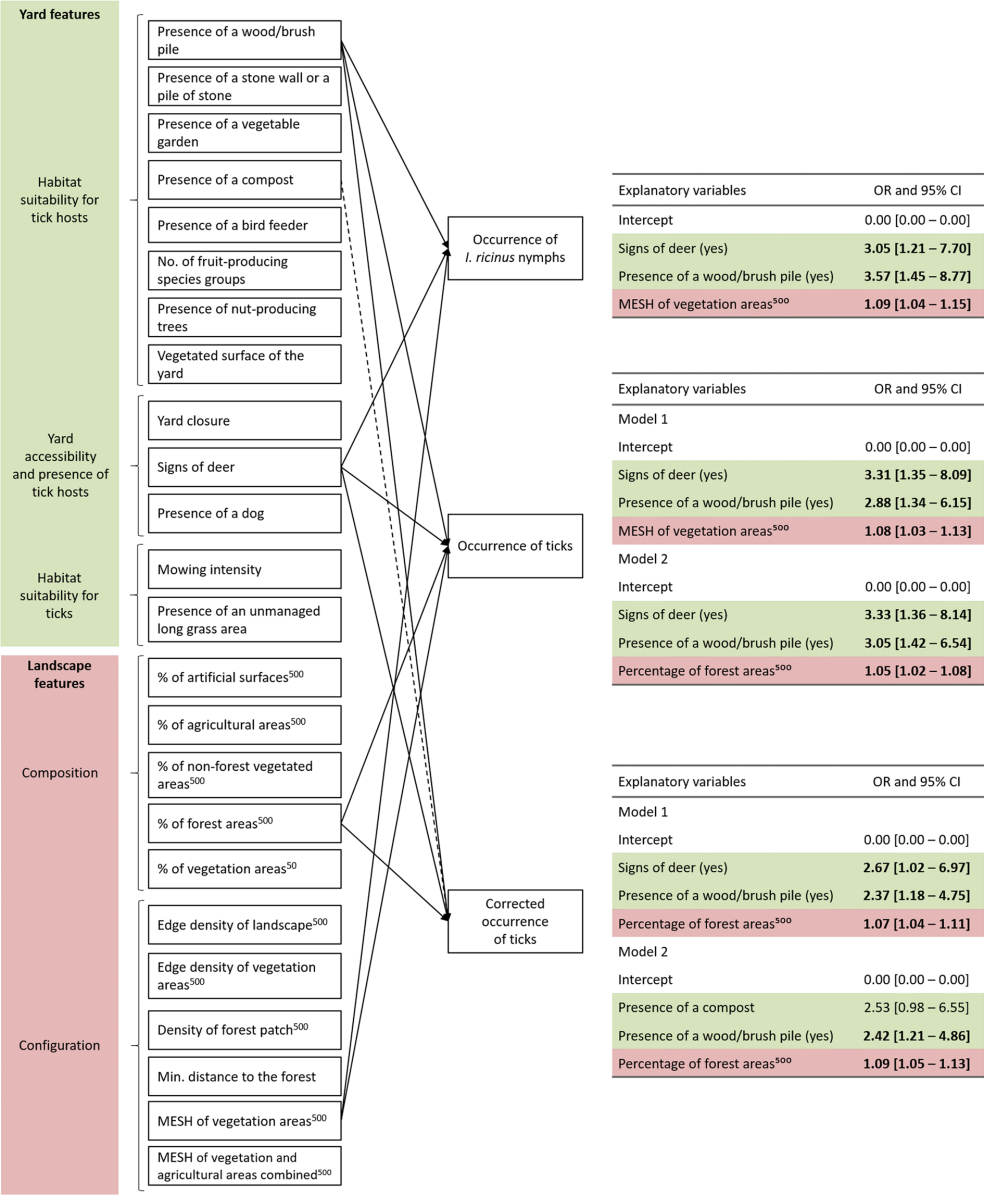
Summary of the best models for risk factors associated with the three occurrence variables. Odd ratios with 95% confidence interval are shown for each explanatory variable. Bold indicates confidence interval excluding one. Non-significant relationships are shown as dotted lines. In model summaries, reference levels are no signs of deer and no wood/brush pile. Variables were computed within a 500-m (^500^) or a 50-m buffer (^50^) from property edges. MESH: Effective mesh size

Nine, twelve, and ten models explaining variations in the occurrence of nymphs, ticks, and corrected occurrence of ticks, respectively, were within ΔAIC < 2 (Tables S4, S5, S6). Among these models, one model best explained the occurrence of *I. ricinus* nymphs (Table S4), while two models best explained the occurrence of ticks and the corrected occurrence of ticks (Tables S5, S6). These models always included three explanatory variables. Overall, the presence of a wood/brush pile and signs of deer in/near the yard were included in all best models (except for a model for signs of deer) and were positively associated with all three occurrence variables (Fig. 2). In yards with a wood/brush pile, the odds of finding ticks or nymphs was 2.37 (95% CI 1.18–4.75) to 3.57 (95% CI 1.45–8.77) greater compared to yards without this feature. Nymphs were observed in 33% of yards with a wood/brush pile compared to 12% of yards without it (Table 3). Similarly, the presence of deer signs in/near the yard increased the odds of finding ticks or nymphs by 2.67 (95% CI 1.02–6.97) to 3.33 (95% CI 1.36–8.14) compared to yards without signs of deer (Fig. 2). Nymphs were observed in 16% of the yard without signs of deer, while in contrast, they were found in 57% of the yard with signs of deer nearby (Table 3).

In addition to being positively associated with signs of deer and the presence of a wood/brush pile, the odds of finding a nymph also increased by 1.09 (95% CI 1.04–1.15) with a one-unit increase in MESH of vegetation areas (Table S4, Fig. S2). Nymphs were observed in 15% of the yard with a MESH of vegetation areas less than 9.6 ha (average) compared to 44% when the MESH of vegetation areas was higher. One of the best models explaining the occurrence of ticks was similar to the best model explaining the occurrence of nymphs as it also included signs of deer, the presence of a wood/brush pile, and the MESH of vegetation areas. The second best model explaining the occurrence of ticks included the percentage of forest areas instead of the MESH of vegetation areas (Table S5, Fig. 2). This model, which included signs of deer, the presence of a wood/brush pile, and the percentage of forest areas, was identical to one of the best models explaining the corrected occurrence of ticks (Table S6, Fig. 2). In these models, the odds of finding ticks or ticks corrected by tick bites increased by 1.05 (95% CI 1.02–1.08) and 1.07 (95% CI 1.04–1.11) with a one-unit increase in the percentage of forest areas (see Fig. S3 for the occurrence of ticks), respectively. In particular, ticks were observed in 20% of yards with less than 10% of the landscape covered by forest areas (i.e. a below-average percentage), while in contrast, they were found in 54% of the yard with an above-average percentage of forest. Finally, the second best model explaining the corrected occurrence of ticks included the presence of a compost, in addition to the presence of a wood/brush pile, and the percentage of forest areas (Fig. 2, Table S6), but its association with the corrected occurrence of ticks was not significant (OR = 2.53, 95% CI 0.98–6.55; Fig. 2).

For the three response variables, the AIC was the lowest for the models including yard and landscape features, intermediate for models with only landscape features, and the highest for models with only yard features. AUC values (0.72–0.84) were similar irrespective of yard and/or landscape features inclusion. Overall, the sensitivity and specificity of the best models was around 0.67 (range 0.49–0.89) and 0.78 (range 0.60–0.88), respectively (Fig. S5). For the occurrence of nymphs*,* the model with yard and landscape features had a similar specificity and sensibility (~ 0.8), while these values differed in models with only yard or landscape features. Differences in specificity and sensitivity were also observed for all models explaining the variation in the occurrence of ticks or the corrected occurrence of ticks with yard features (Fig. S5).

## Discussion

In Europe, there has been limited research on the factors associated with ticks presence or abundance in vegetated areas other than forests (e.g. green spaces including parks [39, 81]), with even fewer studies focusing on private yards [47]. Using citizen science, we assessed, for the first time in France, the occurrence of ticks in private yards within and around a metropolitan community, and investigated the yard and landscape features related to tick presence. Our study reveals that ticks are frequent in private yards, irrespective of whether they are located in densely populated urban areas, urban areas of intermediate density, or thinly populated rural areas, with ticks detected in 32% of the sampled yards and potentially present in 45% of yards when considering collected ticks and reported tick bites. We also demonstrated that their occurrence was linked to both yard and landscape features. Consistent with previous studies conducted in yards in Germany [47] and in urban green spaces in Europe (reviewed in [82]), nymph density in yards was low (mean of 1.6 individuals per 100 m^2^). This suggests that tick presence is primarily due to introductions by hosts that acquired ticks elsewhere (sink populations), and could be sporadic. Nonetheless, 29% of households and 18% of family members reported tick bites in their yard over the last three years, aligning with previous studies highlighting the risk of tick bites in yards [48–51]. Although the participating households were not randomly selected across the study area, which can lead to some biases that limit the representativeness of our study, there was no apparent massive participation of citizens who were previously bitten by ticks in our study. Taken together our findings emphasize the importance of informing the public about the risk of tick exposure and tick bites in yards.

Tick distribution depends on habitat suitability for ticks and their hosts [20, 21]. At the transect level, nymphs were found across all types of transects, regardless of vegetation shading, location, or grass height, indicating their ability to occur in habitats typically deemed less suitable due to less favorable microclimatic conditions. Moreover, in yards with nymphs, the likelihood of finding a nymph was similar in long and short grass areas, as well as in the core or the edge of the yard, whereas it was nearly three times higher in transects beneath trees, hedges, and shrubs compared to transects in open areas without vegetation shading. Compared to open areas, the cover provided by trees and shrubs can buffer temperature extremes and maintain a higher humidity [29], thereby enhancing tick survival [23, 24]. Moreover, trees and shrubs may provide food resources, shelters, breeding, or resting sites for hosts. The abundance or cover of the tree and shrub layer are important predictors of *I. ricinus* nymphs density in various environments, including woodlands [44, 83–86], agricultural areas [87], and urban green spaces [88–90]. However, contrary to our results, Richter et al. [47] found no relationship between *I. ricinus* occurrence in transects and transect shading in yards, suggesting that the effect of vegetation shading might be variable in yards according to other features (type of vegetation, etc.). The lower shading effectiveness of herb layer vegetation compared to trees and shrubs [91] may explain the lack of a relationship between nymph occurrence and grass height (see also: [92]). However, the influence of the herb layer on ticks may be context-dependent (e.g. depending on yard size and availability of suitable habitats nearby), explaining why some studies found an association between tick density and the herb layer (e.g. [88] in urban parks), while others have not (e.g. [87] in pasture edges, [90, 93] in sub-urban and rural parks).

At the yard level, models for the three occurrence variables (i.e. occurrence of nymphs and ticks, and the corrected occurrence of ticks) were similar, given that most ticks were nymphal *I. ricinus*. In all models, the likelihood of finding ticks or nymphs in a yard increased with the presence of a wood/brush pile and signs of deer in/near the yard (Fig. 2). This aligns with previous studies in the US showing a higher occurrence or abundance of three tick species (including *I. scapularis)* in residential properties with a log or brush pile [38, 94] (but see: [95]). Despite the expectation that wood/brush piles attract birds and small mammals, primary hosts for immature *I. ricinus* ticks, studies investigating its influence on tick hosts in yards remain scarce and have shown weak or no relationship with mammal species abundance or richness [96–98]. Therefore, the causal relationships between the presence of a wood/brush pile and tick occurrence should be further investigated. Cervids are vital for the persistence of *I. ricinus* populations, as adult ticks mainly feed and copulate on deer [12]. While cervids may contribute to tick presence in some yards, signs of deer more likely indicate yard proximity to forests. Indeed, the likelihood of observing signs of deer decreased with the minimal distance to the forest (OR = 0.99, 95% CI 0.99–1.0; mean minimal distance to the forest: 121 m [SD = 157 m] for yards with signs of deer vs 357 m [SD = 331 m] for yards without signs of deer). Mowing intensity, the presence of an unmanaged long grass area, a bird feeder, a vegetable garden, and yard closure were yard features absent from the top models (within ΔAIC < 2 from the lowest scoring model). The lack of relationship between the occurrence variables and mowing intensity and the presence of an unmanaged long grass area aligns with our finding at the transect level, where nymph occurrence was not related to grass height. Surprisingly, yard closure (preventing medium and large-sized mammals from entering the yard) was also not related to the occurrence variables (but see: [38] in the USA). This might be explained by the fact that we relied on citizen’s perception and did not accurately estimate fence permeability based on explicit criteria (e.g. fence type, height, and structural integrity). Alternatively, this finding might indicate that ticks are primarily introduced by hosts little or not affected by the most common types of fences in our yards (i.e. hedge and chain link), such as small mammals and birds.

At the landscape scale, we found evidence that the odds of at least one of the three tick occurrence variables were positively linked to the percentage of forest areas (tick occurrence and corrected occurrence of ticks) and the MESH of vegetation areas (occurrence of nymphs and corrected occurrence of ticks). This finding aligns with previous literature indicating that, in Europe, forests are more suitable habitats for ticks compared to non-forest land-use types [37]. As previously shown for tick persistence in meadows, forests can also be considered as a source of ticks for yards, while more artificialized or open landscape types (e.g. crops, artificial surfaces, or even yard lawns) act as sinks [99]. The positive association between the MESH of vegetation areas (i.e. a decrease in the fragmentation) and the occurrence variables is unsurprising considering the positive correlation between the MESH of vegetation areas and the percentage of forest areas (r = 0.66, Fig. S4). The importance of landscape connectivity for tick populations has already been identified in rural areas in Spain [41, 42], as well as in green spaces in urban areas in Belgium [39], and the US [38, 40]. Given favorable conditions for tick survival in yards, enhanced connectivity between suitable habitats for ticks, their hosts, and potential source host populations (e.g. forest) could promote tick presence in yards and thus poses a potential public health risk.

Citizen science is a valuable approach for advancing ecological knowledge, yet concerns persist regarding data quality [53, 100]. We implemented several recommended measures to ensure data quality and reliability [101], including a standardized protocol for tick collection, available in written and video format, as well as a support for tick recognition. Additionally, we asked participants to record easily identifiable yard characteristics. Although we did not quantify it, the participation of citizens in this study likely increased their tick risk awareness and tick-associated literacy, based on their feedbacks. This may in turn increase the acceptance of methods to prevent tick bites (but see: [102]).

One of the objectives of our project was to determine whether certain features of the yard or landscape which can be modified to reduce the risk of tick occurrence. However, our results should be interpreted with caution regarding public health. For example, tick occurrence was related to the percentage of forest areas in the landscape and the connectivity of vegetated areas. This suggests that initiatives aimed at enhancing landscape connectivity, and/or creating vegetated areas near yards may increase tick exposure risk, but final decisions must be balanced against other benefits, such as an increase in biodiversity or human well-being [103]. Additionally, tick occurrence was linked to vegetation shading, the presence of a wood/brush pile, and signs of deer in/near the yard. However, we caution against providing clear recommendations to yard owners based solely on these results. Indeed, further research is needed to establish causal relationships between these features and tick occurrence, as well as to assess the potential impact of yard management measures on fauna and the associated financial implications for yard owners. Nonetheless, yard owners should be particularly vigilant when using shaded areas (e.g. while trimming hedges or lying down a tree), as nymph occurrence was three times higher than in unshaded areas. In our study the risk of encountering ticks did not appear to be associated with the presence of a stone wall, a vegetable garden, a compost, a bird feeder, a dog, and nut-producing trees, the vegetated surface of the yard, yard closure, mowing intensity, grass height, and the number of fruit-producing trees. Moreover, it was similar in the core and the edge of the yard. Health authorities (e.g. USA and Canada) have recommended various property management measures to control tick populations (e.g. keep grass mowed and move bird feeders away from the house [104]), not supported by the results of this study, but often cited in scientific or popular science publications (e.g. [105, 106]). As previously stated [95], we recommend that public health officials provide clear information on the scientific support and uncertainties surrounding yard management measures, as most of them seem to lack scientific support. European health authorities should also be careful when using results from North American studies, as yard features (especially the size), tick species, and host ecology, might differ greatly from European yards. Further studies are necessary to identify features associated with tick occurrence in yards and to implement management strategies adapted to the private yards to mitigate the risk of tick exposure.

## Conclusions

Ticks are frequent in private yards across an urbanization gradient in our study area in northeastern France and their presence is shaped by both yard and landscape features. Despite official recommendations on some yard measures expected to control ticks abundance or occurrence, we found no evidence linking *I. ricinus* occurrence in yards with the mowing intensity and other relevant yard features. However, we found that vegetation shading, the presence of a wood/brush pile, and signs of deer in/near the yard were related to the occurrence of ticks. At the landscape level, tick occurrence were positively related to the percentage of forest areas, and the connectivity of vegetated areas. We suggest further studies are needed to implement adapted management strategies against tick risk in yards. To better understand the threat posed by ticks in yards and enhance management measures effectiveness, future research should investigate the prevalence of pathogens in the ticks present in yards, as well as identify the hosts responsible for tick presence in yards.

## Supplementary Information

Supplementary Material 1.

## Data Availability

The data used in this paper includes personal residential information. Therefore, to protect the privacy of the study participants, the data are not publicly available. The anonymized data collected are, however, available from the corresponding author upon reasonable request.
