# Supplementary material for: Understanding Ixodes ricinus occurrence in private yards: influence of yard and landscape features
Source: Int J Health Geogr. 2024 Oct 10;23:21. doi: 10.1186/s12942-024-00380-9 (PMC11468097; doi:10.1186/s12942-024-00380-9)
Supplement: Supplementary file 1 — Supplementary Material 1. [file 12942_2024_380_MOESM1_ESM.docx]

**Supplementary material S1: Standardized tick sampling protocol and full description of the content of the ‘tick collection kit’**

The protocol and the questionnaires were co-constructed between scientists specialized in ticks and/or citizen science, and science communicators and educators from a local environmental education association. Both documents were slightly improved in their formulation based on feedbacks from citizens who participated during the first campaign (i.e. in 2020). ‘Tick collection kit’ content and protocol were designed to improve the tick literacy of citizens and facilitate protocol appropriation and realization.

Interested citizen scientists had to pick up a ‘tick collection kit’ from one of the project partners (between one and four depending on the year). This kit, identified by a unique ID, included a 1 m^2^ white cloth suspended by a string from a PVC pipe for finding ticks, a magnifying glass and dissecting tweezers to identify and handle ticks, and 12 2-ml Eppendorf tubes filled with 70% ethanol (numbered from 1 to 12) to store ticks. We also provided data sheets and a printed version of the sampling protocol that detailed the content of the kit and explained how to select appropriate sampling sites, collect ticks, and fill out the data sheets. To increase participants’ knowledge about ticks and to teach them to distinguish ticks from insects and spiders and determine the species of tick, we created two summarized identification keys and gave them a vial with *Ixodes ricinus* preserved in ethanol. The vials always contained a nymph and most of them also contained a larva and an adult. Additionally, a 7-minute video that detailed the content of the kit and the sampling protocol was available on the project website with other documents in PDF (available in French at https://www.citique.fr/tiquojardin-phase-1/). Finally, we supplied a tick remover and prevention documents to raise awareness of the risk of tick bites.

**Table S1: Grouping of trees and bushes/shrubs according to their harvest period in France.**

| Type of vegetation | Species group | Harvest period |
| --- | --- | --- |
| Tree | Cherry tree | Late spring and summer |
| Bush/shrub | Raspberry bush, gooseberry, blackcurrant | Late spring and summer |
| Tree | Plum and peach tree | Summer |
| Bush/shrub | Mulberry and blackberry | Late summer |
| Tree | Apple, pear, and quince tree | Late summer and autumn |
| Bush/shrub | Vine | Late summer and autumn |

**Table S2: Percentage of yards with each tick species, stage, and sex per sampling year.**

| Species | Stage and sex | 2020 | 2021 | 2022 |
| --- | --- | --- | --- | --- |
| *Ixodes ricinus* | larvae | 4% (1) | 5% (3) | 4% (4) |
| *Ixodes ricinus* | nymph | 19% (5) | 34% (20) | 19% (19) |
| *Ixodes ricinus* | adult female | 4% (1) | 5% (3) | 2% (2) |
| *Ixodes ricinus* | adult male | 4% (1) | 8% (5) | 2% (2) |
| *Ixodes frontalis* | larvae | 15% (4) | 0% (0) | 0% (0) |
| *Ixodes frontalis* | nymph | 0% (0) | 2% (1) | 4% (4) |
| *Dermacentor marginatus* | adult female | 0% (0) | 0% (0) | 1% (1) |
| *Dermacentor reticulatus* | adult female | 0% (0) | 2% (1) | 0% (0) |
| *Dermacentor reticulatus* | adult male | 0% (0) | 3% (2) | 1% (1) |
|  | Percentage of yards with ticks (number) | 35% (9) | 41% (24) | 26% (26) |

The number in brackets represents the number of yards in which ticks occurred. Tick collection took place in 185 yards from May to mid-July 2020 (n = 26), 2021 (n = 59), and 2022 (n = 100) within a 35 km radius of Nancy, a city located in the northeast of France.

**Table S3: Relationships between the occurrence of *I. ricinus* nymphs and transect features.**

| Transect feature | Factor levels | No. of transects | Freq. of transect with nymphal *I. ricinus* (number) | OR and 95% CI |
| --- | --- | --- | --- | --- |
| Grass height | <10 cm | 239 | 28% (68) | Reference level |
|  | > 10 cm | 136 | 25% (34) | 1.05 [0.54 – 2.01] |
|  | mixed | 59 | 29% (17) | 0.87 [0.38 – 1.96] |
| Location | core | 284 | 24% (68) | Reference level |
|  | edge | 150 | 34% (51) | 1.52 [0.91 – 2.54] |
| Shading | not shaded | 172 | 17% (30) | Reference level |
|  | shaded | 262 | 34% (89) | 2.75 [1.52 – 4.97] |

This analysis was conducted in yards with nymphal *I. ricinus,* for which information about the transect grass height, location, shading, and length was available for all transects (n = 434 transects in 37 yards).

**Table S4: Models predicting the occurrence of *I. ricinus* nymphs in function of yard and landscape features.**

| Yard features | | | | | | Landscape features | | | | Model selection criteria | | |
| --- | --- | --- | --- | --- | --- | --- | --- | --- | --- | --- | --- | --- |
| Signs of deer | Presence of a dog | No. of fruit-producing species groups | Presence of a compost | Presence of a wood/brush pile | Vegetated surface of the yard | % of agricultural areas⁵⁰⁰ | % of artificial surfaces⁵⁰⁰ | MESH of vegetation areas⁵⁰⁰ | MESH of vegetation and agricultural areas combined⁵⁰⁰ | No. of explanatory variables | AIC | ΔAIC |
| X | X |  |  | X |  |  |  | X |  | 4 | 157.92 | 0.00 |
|  |  | X |  | X |  | X |  |  | X | 4 | 158.67 | 0.74 |
| X |  | X |  | X |  |  |  | X |  | 4 | 158.91 | 0.98 |
| X |  |  | X | X |  |  |  | X |  | 4 | 159.10 | 1.18 |
| X |  |  |  | X | X |  |  | X |  | 4 | 159.22 | 1.29 |
| X |  | X |  |  |  | X |  |  | X | 4 | 159.25 | 1.33 |
| X |  |  |  | X |  | X |  |  | X | 4 | 159.29 | 1.36 |
| X |  |  |  | X |  | X | X |  |  | 4 | 159.59 | 1.66 |
| **X** |  |  |  | **X** |  |  |  | **X** |  | **3** | **159.67** | **1.75** |

Only top candidate models within ΔAIC<2 are presented. The Akaike Information Criterion (AIC) and the differences in AIC from the lowest-scoring model are shown for each model. Bold text indicates the selected model. Variables were computed within a 500-m (^500^) or a 50-m buffer (^50^) from property edges. MESH: Effective mesh size.

**Table S5: Models predicting the occurrence of ticks in function of yard and landscape features.**

| Yard features | | | | |  | Landscape features | | | | | | Model selection criteria | | |
| --- | --- | --- | --- | --- | --- | --- | --- | --- | --- | --- | --- | --- | --- | --- |
| Signs of deer | Presence of a dog | Presence of a stone wall or a pile of stone | Presence of a wood/brush pile | Vegetated surface of the yard | Avg. daily saturation deficit | % of agricultural areas⁵⁰⁰ | % of non-forest vegetated areas⁵⁰⁰ | % of forest areas⁵⁰⁰ | Min. distance to the forest | Density of forest patch | MESH of vegetation areas⁵⁰⁰ | No. of explanatory variables | AIC | ΔAIC |
| X |  |  | X | X |  |  |  |  |  |  | X | 4 | 193.21 | 0.00 |
| X |  |  | X |  |  |  |  |  | X |  | X | 4 | 193.75 | 0.55 |
| X |  | X | X |  |  |  |  | X |  |  |  | 4 | 193.83 | 0.63 |
| X |  |  | X |  |  |  | X | X |  |  |  | 4 | 194.10 | 0.90 |
| X |  |  | X |  |  | X |  |  |  |  | X | 4 | 194.34 | 1.13 |
| X |  | X | X |  |  |  |  |  |  |  | X | 4 | 194.44 | 1.24 |
| X |  |  | X |  |  |  |  |  |  | X | X | 4 | 194.76 | 1.56 |
| X |  |  | X |  | X |  |  | X |  |  |  | 4 | 194.88 | 1.68 |
| **X** |  |  | **X** |  |  |  |  |  |  |  | **X** | **3** | 194.96 | 1.75 |
| **X** |  |  | **X** |  |  |  |  | **X** |  |  |  | **3** | 194.96 | 1.76 |
| X | X |  | X |  |  |  |  |  |  |  | X | 4 | 195.04 | 1.83 |
| X | X |  | X |  |  |  |  | X |  |  |  | 4 | 195.08 | 1.88 |

Only top candidate models within ΔAIC<2 are presented. The Akaike Information Criterion (AIC) and the differences in AIC from the lowest-scoring model are shown for each model. Bold text indicates the selected model. Variables were computed within a 500-m (^500^) or a 50-m buffer (^50^) from property edges. MESH: Effective mesh size.

**Table S6: Models predicting the corrected occurrence of ticks in function of yard and landscape features.**

| Yard features | | | | | | Landscape features | | | | | Model selection criteria | | |
| --- | --- | --- | --- | --- | --- | --- | --- | --- | --- | --- | --- | --- | --- |
| Signs of deer | Presence of a wood/brush pile | Presence of a compost | No. of fruit-producing species group | Presence of nut-producing trees | Vegetated surface of the yard | % of agricultural areas⁵⁰⁰ | % of forest areas⁵⁰⁰ | % of non-forest vegetated areas⁵⁰⁰ | % of vegetation areas⁵⁰ | MESH of vegetation and agricultural areas combined⁵⁰⁰ | No. of explanatory variables | AIC | ΔAIC |
| X | X | X |  |  |  |  | X |  |  |  | 4 | 212.19 | 0.00 |
| **X** | **X** |  |  |  |  |  | **X** |  |  |  | **3** | **213.47** | **1.28** |
|  | X | X |  |  |  |  | X |  | X |  | 4 | 213.48 | 1.29 |
|  | X | X |  |  |  |  | X |  |  | X | 4 | 213.57 | 1.39 |
|  | **X** | **X** |  |  |  |  | **X** |  |  |  | **3** | **213.70** | **1.51** |
| X | X |  |  |  | X |  | X |  |  |  | 4 | 213.75 | 1.57 |
| X | X |  | X |  |  |  | X |  |  |  | 4 | 213.76 | 1.57 |
|  |  |  | X |  |  | X | X |  |  | X | 4 | 213.97 | 1.79 |
|  |  |  | X |  |  | X |  | X |  | X | 4 | 213.98 | 1.79 |
| X | X |  |  | X |  |  | X |  |  |  | 4 | 214.04 | 1.85 |

Only top candidate models within ΔAIC<2 are presented. The Akaike Information Criterion (AIC) and the differences in AIC from the lowest-scoring model are shown for each model. Bold text indicates the selected model. Variables were computed within a 500-m (^500^) or a 50-m buffer (^50^) from property edges. MESH: effective mesh size.

**Table S7: Comparisons of the best models predicting the occurrence of *I. ricinus* nymphs.**

|  | Yard features | | Landscape features | | | Model accuracy metrics |
| --- | --- | --- | --- | --- | --- | --- |
| Model | Signs of deer | Presence of a wood/brush pile | % of artificial surfaces⁵⁰⁰ | MESH of vegetation areas⁵⁰⁰ | MESH of vegetation and agricultural areas combined⁵⁰⁰ | AIC |
| Y + L | X | X |  | X |  | 159.67 |
| L |  |  | X |  | X | 168.33 |
| Y | X | X |  |  |  | 173.11 |

The best models included only yard features (Y), landscape features (L), or both features (Y + L). Variables were computed within a 500-m (^500^) or a 50-m buffer (^50^) from property edges. MESH: effective mesh size.

**Table S8: Comparisons of the best models predicting the occurrence of ticks.**

|  | Yard features | | Landscape features | | | | | | Model accuracy metrics |
| --- | --- | --- | --- | --- | --- | --- | --- | --- | --- |
| Model | Signs of deer | Presence of a wood/brush pile | % of forest areas⁵⁰⁰ | % of agricultural areas⁵⁰⁰ | % of vegetation areas⁵⁰ | Min. distance to the forest | MESH of vegetation areas⁵⁰⁰ | MESH of vegetation and agricultural areas combined⁵⁰⁰ | AIC |
| Y + L | X | X |  |  |  |  | X |  | 194.96 |
| Y + L | X | X | X |  |  |  |  |  | 194.96 |
| L |  |  |  | X |  | X |  | X | 202.56 |
| L |  |  |  | X |  | X | X |  | 203.79 |
| L |  |  |  | X | X |  |  | X | 204.04 |
| Y | X | X |  |  |  |  |  |  | 205.36 |

The best models included only yard features (Y), landscape features (L), or both features (Y + L). Variables were computed within a 500-m (^500^) or a 50-m buffer (^50^) from property edges. MESH: effective mesh size.

**Table S9: Comparisons of the best models predicting the corrected occurrence of ticks.**

|  | Yard features | | | | Landscape features | | | | | | | | Model accuracy metrics |
| --- | --- | --- | --- | --- | --- | --- | --- | --- | --- | --- | --- | --- | --- |
| Model | Signs of deer | Presence of a wood/brush pile | Presence of a compost | Presence of nut-producing trees | % of forest areas⁵⁰⁰ | % of agricultural areas⁵⁰⁰ | % of artificial areas⁵⁰⁰ | % of non-forest vegetated areas⁵⁰⁰ | MESH of vegetation areas⁵⁰⁰ | MESH of vegetation and agricultural areas combined⁵⁰⁰ | % of vegetation areas⁵⁰ | Min. distance to the forest | AIC |
| Y + L | X | X |  |  | X |  |  |  |  |  |  |  | 213.47 |
| Y + L |  | X | X |  | X |  |  |  |  |  |  |  | 213.70 |
| L |  |  |  |  |  | X |  | X |  | X | X |  | 215.51 |
| L |  |  |  |  |  | X |  | X |  | X |  | X | 216.92 |
| L |  |  |  |  |  | X | X | X | X |  |  |  | 217.22 |
| Y | X | X |  |  |  |  |  |  |  |  |  |  | 233.55 |
| Y | X |  |  | X |  |  |  |  |  |  |  |  | 234.78 |

The best models included only yard features (Y), landscape features (L), or both features (Y + L). Variables were computed within a 500-m (^500^) or a 50-m buffer (^50^) from property edges. MESH: effective mesh size.


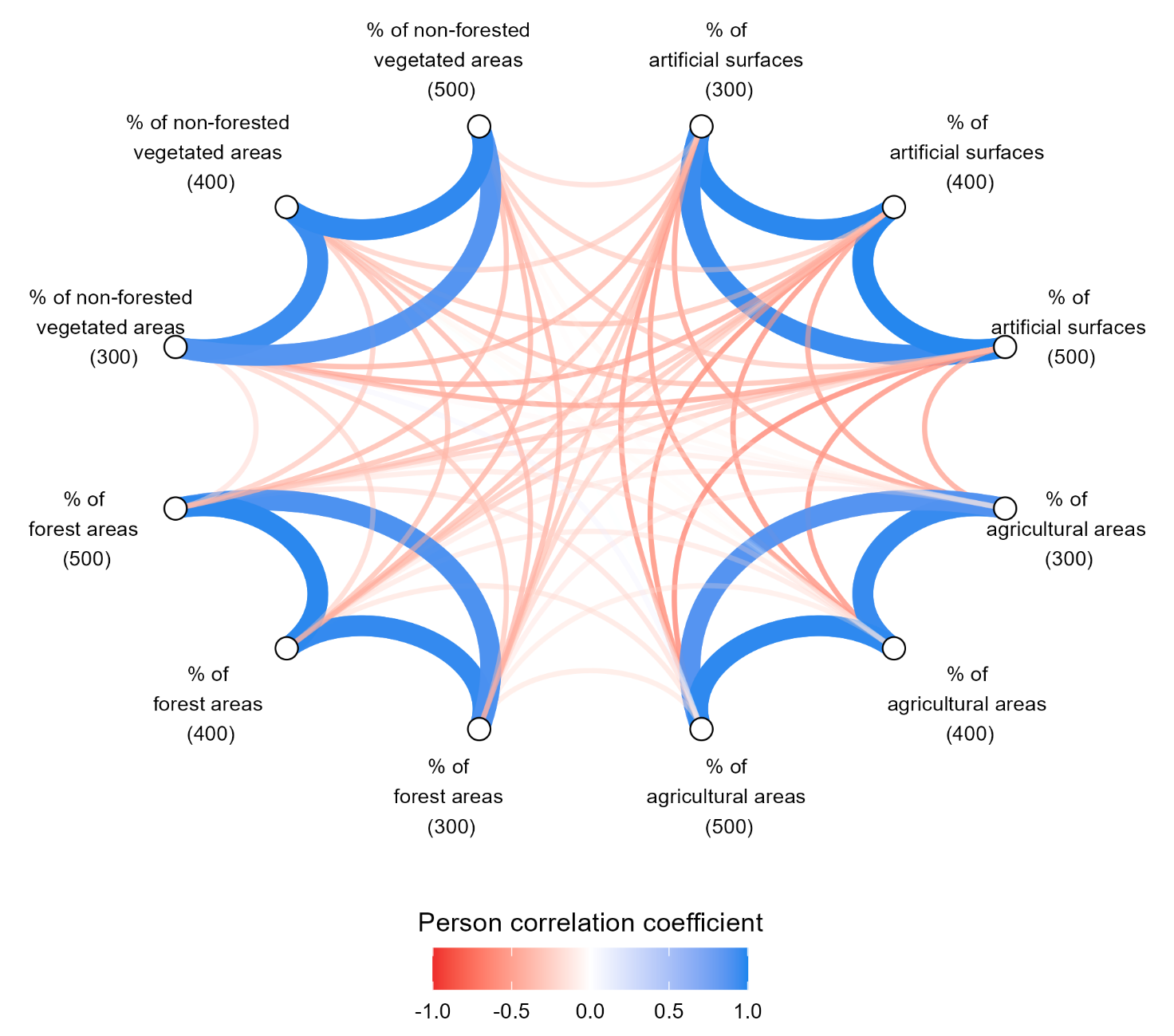


**Figure S1: Pearson correlation coefficients for the landscape features computed at three buffer sizes.** Thin lines indicate a |Pearson correlation coefficient| < 0.6 while thick lines indicate a |Pearson correlation coefficient| ≥ 0.6. The color intensity reflects the strength of the correlation. Variables were computed within a 300-m buffer (300), a 400-m buffer (400), or a 500-m buffer (500) from the edges of the property.


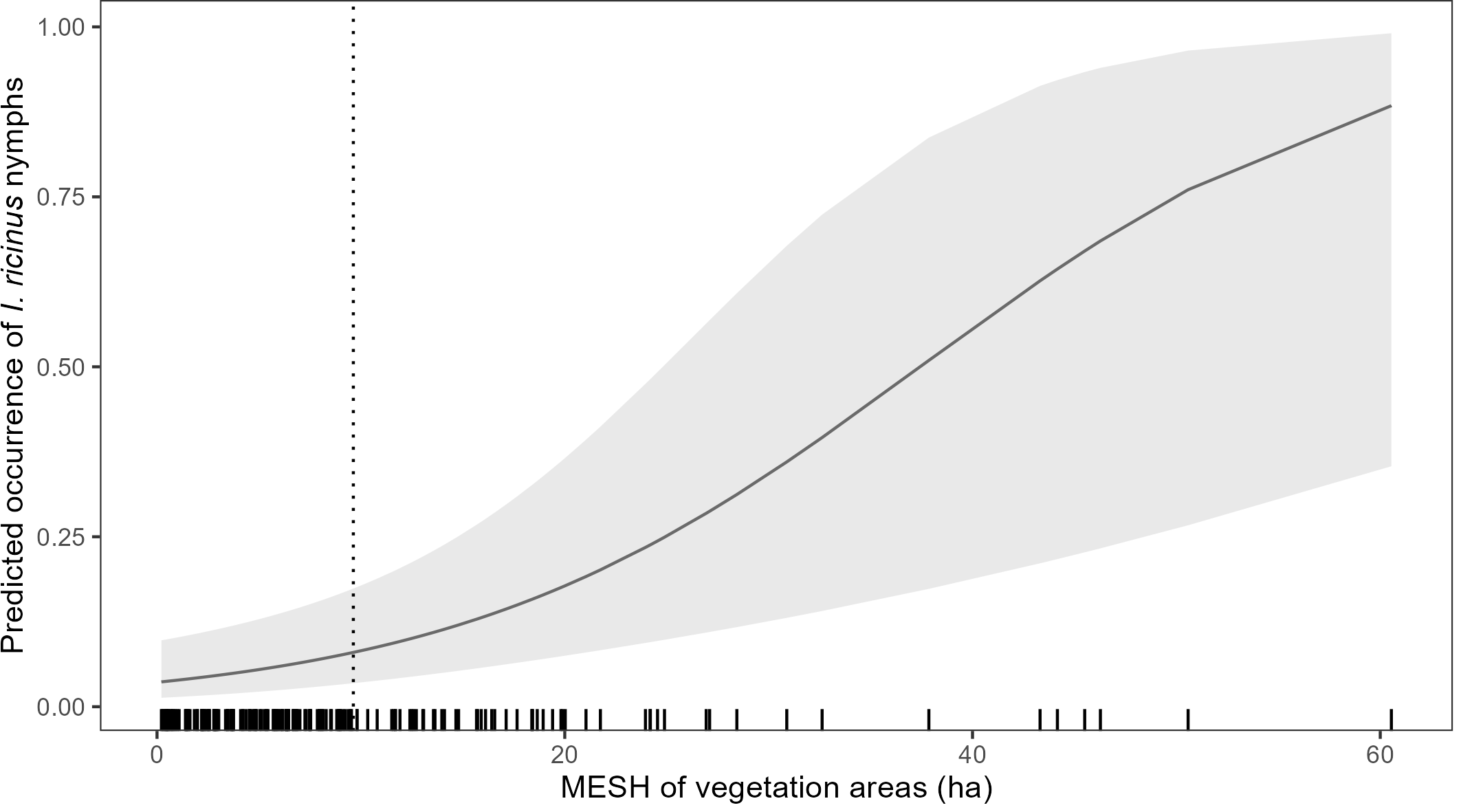


**Figure S2: Predicted occurrence of *I. ricinus* nymphs in function of the MESH of vegetation areas.** The shaded area represents the 95% CI around the predicted values (solid line). The predicted values are computed for yards with no sign of deer, no wood/brush pile, and a sampled surface of 100 m^2^. The rug represents observed values. The dotted line represents the average MESH of vegetation areas in yards (9.6 ha). MESH: effective mesh size.


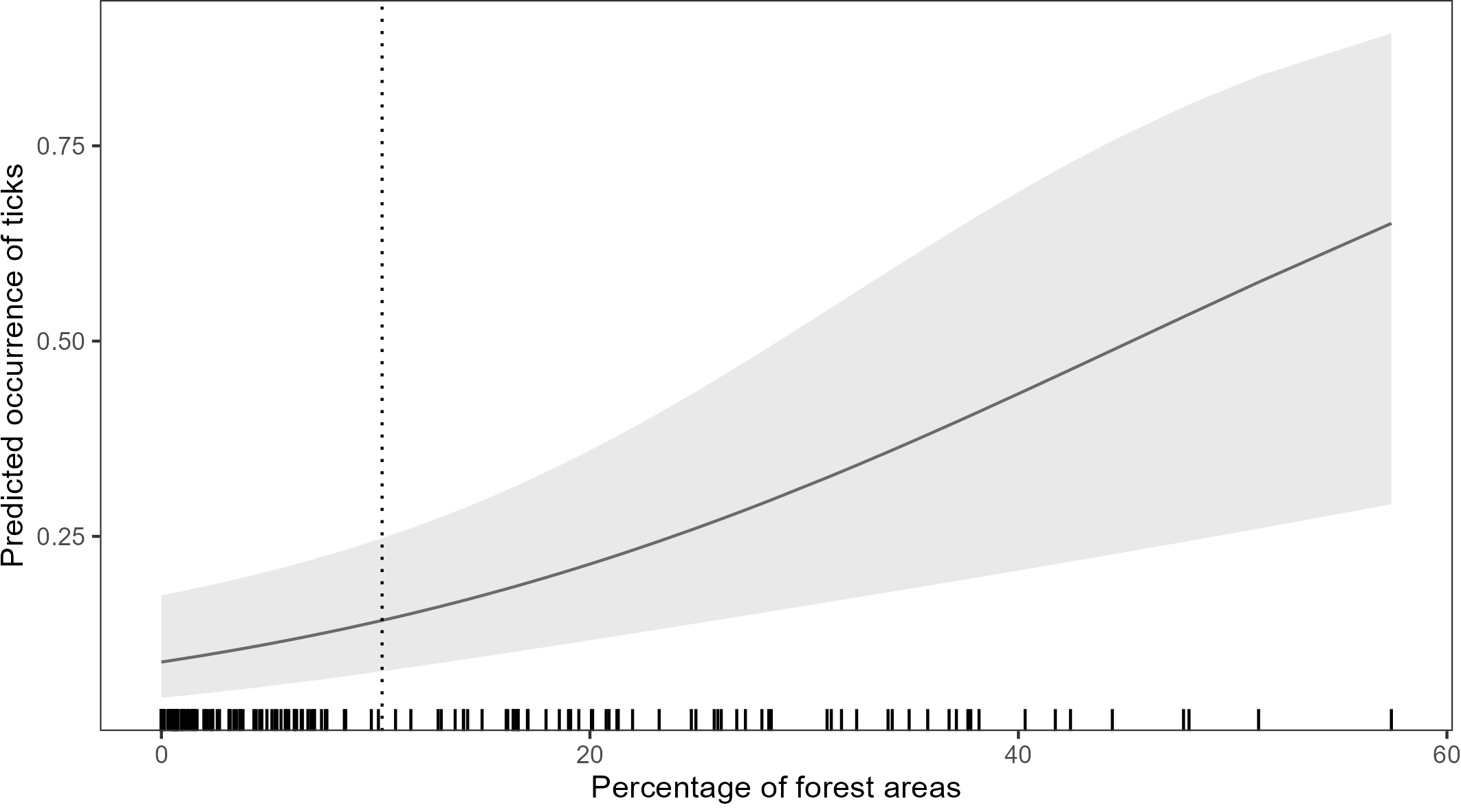


**Figure S3: Predicted occurrence of ticks in function of the percentage of forest areas.** The shaded area represents the 95% CI around the predicted values (solid line). The predicted values are computed for yards with no sign of deer, no wood/brush pile, and a sampled surface of 100 m^2^. The rug represents observed values. The dotted line represents the average percentage of forest areas around yards (10%).


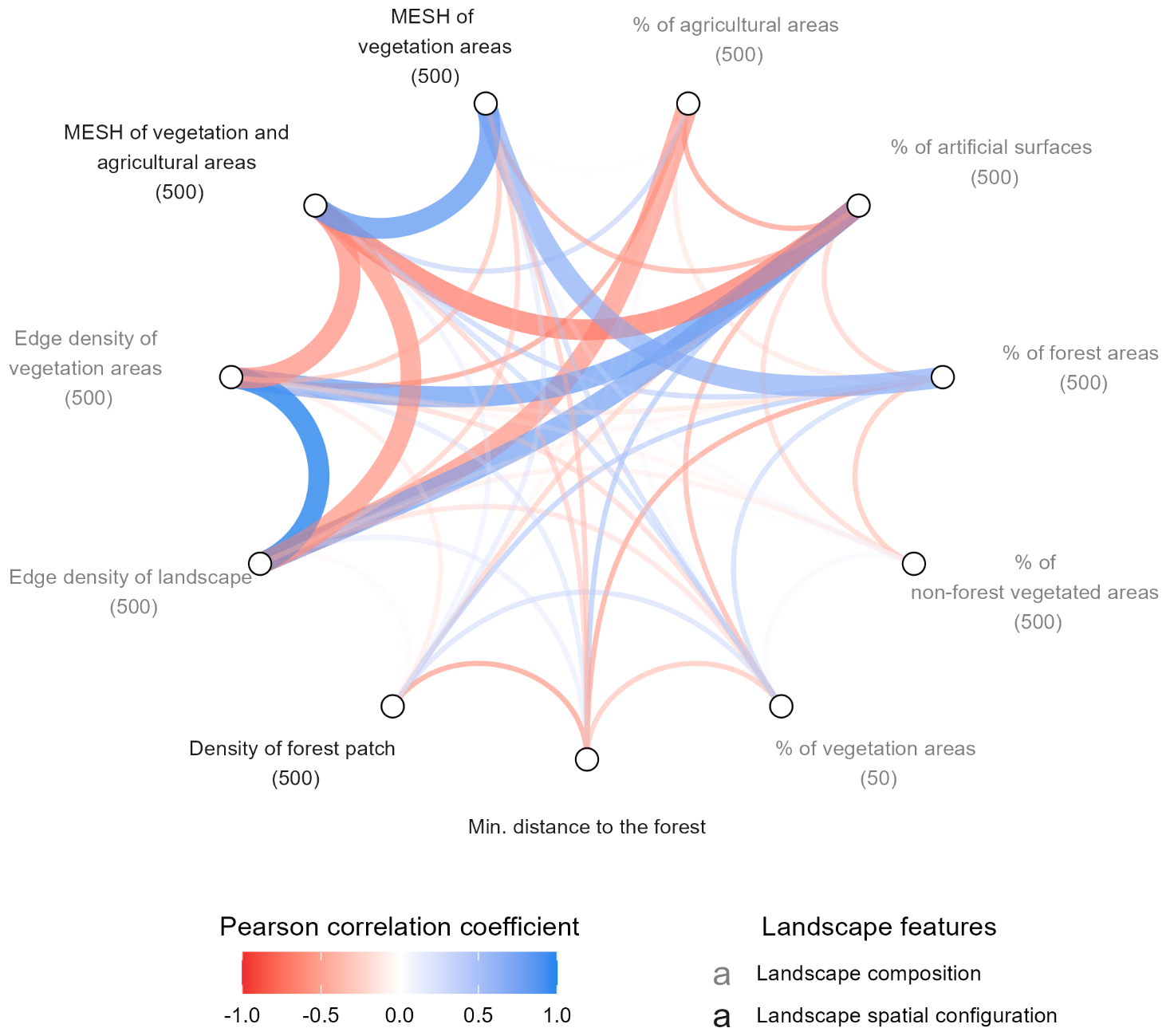


**Figure S4: Pearson correlation coefficient for the features describing the landscape composition and spatial configuration.** These variables were included in the models to explore the relationships between environmental factors and the occurrence of nymphal *I. ricinus,* ticks, and the corrected occurrence of ticks. Thin lines indicate a |Pearson correlation coefficient| <0.6 while thick lines indicate a |Pearson correlation coefficient| ≥ 0.6. The color intensity reflects the strength of the correlation.


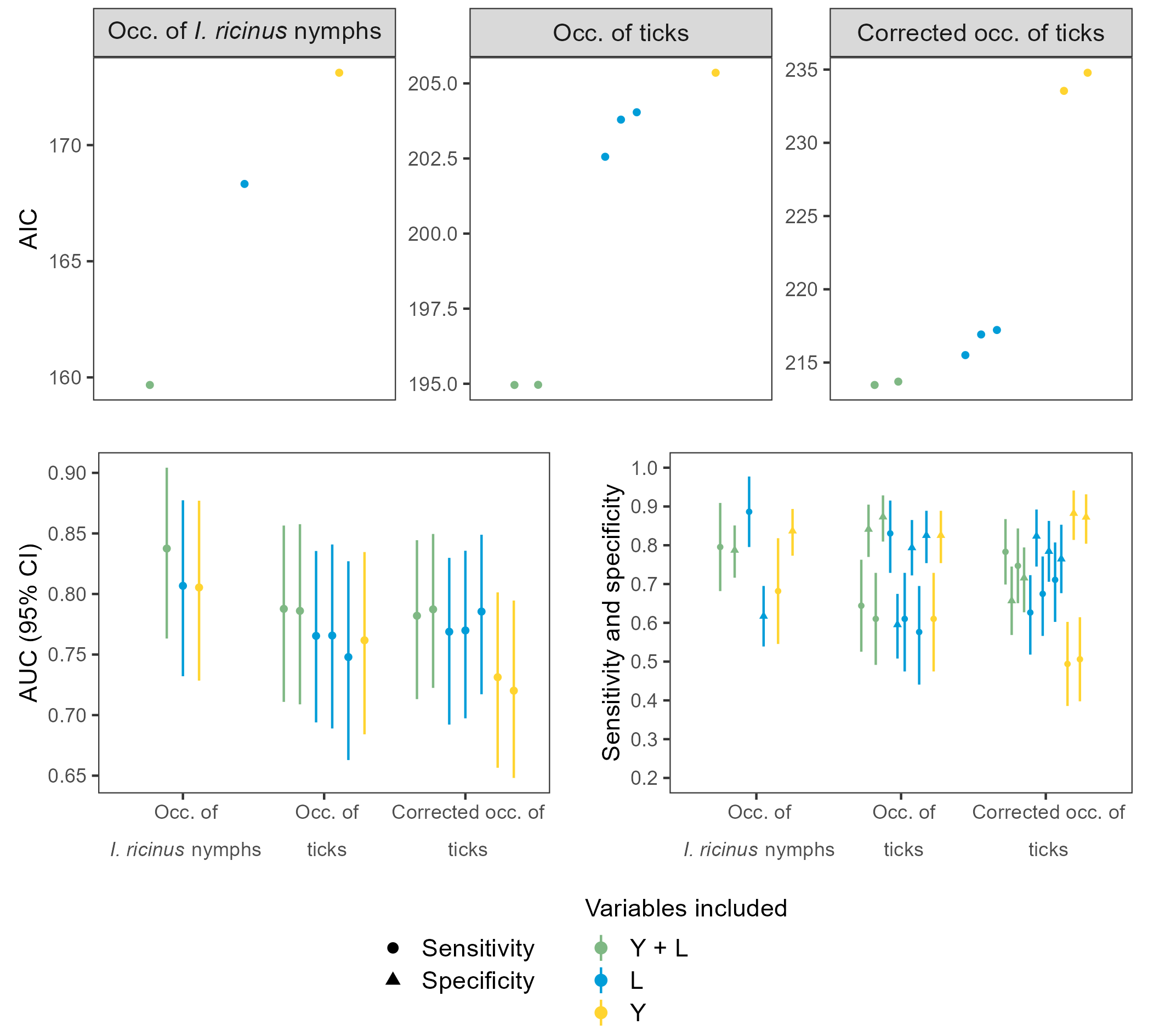


**Figure S5: AIC, AUC, sensibility, and specificity for the best models explaining the three occurrence variables.** The sensibility and specificity were computed at the optimal cutpoint value that maximizes them (Youden index [70]). 95% CI around the accuracy metrics are shown.
